# Trends in Vibrational Spectroscopy: NIRS and Raman Techniques for Health and Food Safety Control

**DOI:** 10.3390/s26030989

**Published:** 2026-02-03

**Authors:** Candela Melendreras, Jesús Montero, José M. Costa-Fernández, Ana Soldado, Francisco Ferrero, Francisco Fernández Linera, Marta Valledor, Juan Carlos Campo

**Affiliations:** 1 Department of Physical and Analytical Chemistry, University of Oviedo, 33006 Oviedo, Asturias, Spain; 2 Department of Electrical, Electronics, Communications and Systems Engineering, University of Oviedo, 33204 Gijón, Asturias, Spain

**Keywords:** non-invasive analysis, real-time analysis, near-infrared spectroscopy (NIRS), Raman spectroscopy, chemometrics analysis, hyperspectral imaging (HSI), partial least squares (PLS), principal component analysis (PCA)

## Abstract

There is an increasing need to establish reliable safety controls in the food industry and to protect public health. Consequently, there are numerous efforts to develop sensitive, robust, and selective analytical strategies. As regulatory requirements for food and the concentration for target biomarkers in clinical analysis evolve, the food and health sectors are showing a growing interest in developing non-destructive, rapid, on-site, and environmentally safe methodologies. One alternative that meets the conditions is non-destructive spectroscopic sensors, such as those based on vibrational spectroscopy (Raman, surface-enhanced Raman—SERS, mid- and near-infrared spectroscopy, and hyperspectral imaging built on those techniques). The use of vibrational spectroscopy in food safety and health applications is expanding rapidly, moving beyond the laboratory bench to include on-the-go and in-line deployment. The dominant trends include the following: (1) the miniaturisation and portability of instruments; (2) surface-enhanced Raman spectroscopy (SERS) and nanostructured substrates for the detection of trace contaminants; (3) hyperspectral imaging (HSI) and deep learning for the spatial screening of quality and contamination; (4) the stronger integration of chemometrics and machine learning for robust classification and quantification; (5) growing attention to calibration transfer, validation, and regulatory readiness. These advances will bring together a variety of tools to create a real-time decision-making system that will address the issue in question. This article review aims to highlight the trends in vibrational spectroscopy tools for health and food safety control, with a particular focus on handheld and miniaturised instruments.

## 1. Introduction

Food safety assurance and clinical parameters for monitoring are two critical aspects that intersect in safeguarding human health and well-being. Ensuring food safety is crucial for protecting public health and for instilling consumer confidence. It involves implementing rigorous standards and control measures to prevent foodborne illnesses and hazards throughout the production chain. Regarding clinical parameters monitoring, they play a pivotal role in disease prevention, diagnosis, and management. Regular assessments of vital signs, blood chemistry, and other health indicators allow healthcare professionals to identify the early warning signs of illness, to track the progression of diseases, and to evaluate the effectiveness of treatment interventions. By closely monitoring these parameters, individuals can take proactive steps to maintain their health and wellness, make informed lifestyle choices, and seek timely medical intervention when necessary. Many processes have been described to achieve the required clinical or food information. However, vibrational spectroscopy is one of the most powerful techniques that can give a real-time and/or non-invasive response to solve the analytical approach.

Over the last few decades, vibrational spectroscopy has established itself as a useful and applicable technology in a wide range of sectors, including agri-food, pharmaceuticals, petrochemicals and healthcare. As the requirements of each sector change over time, it has been recognised that these techniques have attracted even more interest as part of the suite of non-destructive spectral sensors (NDSSs). These spectroscopic technologies, coupled with data analysis and chemometrics, have allowed the development of new trends towards non-target methods that can be used to analyse products and to create a unique fingerprint to provide information on food safety and health parameters. Furthermore, vibrational spectroscopy, when combined with data analysis, offers cost-effective, value-added solutions to a range of food industry and health problems. NIR, Raman, and FTIR spectroscopy represent key vibrational techniques, each offering distinct analytical capabilities that enhance molecular and structural studies. Raman spectroscopy is based on scattering, whereas the NIR spectrum is due to the absorption of radiation. Both can be used for simple spectrum measures or for acquiring images by scanning simultaneously image information and spectra. Table 1 details the similarities and differences between the two spectroscopic techniques.

Raman and NIR spectroscopy are the most attractive techniques for the agri-food and health sector, due to their simplicity, ease of use, and multi-parametric character, broadening the range of sample analysis to facilitate a comprehensive investigation. In this manuscript, the main trends in the development of methodologies based on vibrational spectroscopy (NIR and Raman spectroscopy) applied to food safety and medicine will be presented, including conventional spectroscopy, imaging and microscopy proposals, instrumental trends (focusing on miniaturised and portable instruments), as well as new strategies and trends in chemometric approaches.

As the analytical needs of the food and health sectors evolve, several dominant trends are shaping the development and deployment of vibrational spectroscopy tools. These trends form the backbone of the present review and are addressed in dedicated sections.

Section 2 examines the miniaturisation and portability of vibrational spectroscopy instruments, including MEMS-based architecture, handheld NIR devices, and fibre-optic probes. Section 3 focuses on advances in Raman technologies, with particular emphasis on SERS substrates, SORS, Raman imaging, and the growing availability of portable Raman systems. Section 4 explores the rapid expansion of hyperspectral imaging (HSI) and the integration of deep learning for spatial–spectral analysis in food and biomedical applications. Section 5 reviews the increasing role of chemometrics and machine learning, highlighting both classical and modern modelling strategies, as well as cloud based and IoT enabled workflows. Section 6 focus on vibrational spectroscopy applications in food authentication and in health control using NIRS and Raman. Section 7 discusses calibration transfer, validation, and regulatory readiness, which are essential for translating laboratory developed methods into robust, field deployable solutions. Finally, Section 8 presents the conclusions and future perspectives.

The purpose of this review is not only to describe technological evolution but to illustrate how these advances are being translated into practical solutions for food safety control and clinical diagnostics. By structuring the manuscript around five dominant trends, we aim to provide a coherent and accessible overview of the current landscape and future directions of vibrational spectroscopy for health and food safety applications.

## 2. Miniaturisation and Portability of Vibrational Spectroscopy Instruments 

The first major trend shaping the evolution of vibrational spectroscopy is the rapid miniaturisation of instruments [1,2] and the design of multi-mode and single-mode fibre optics [3]. As analytical needs shift towards in-field, in-line, and point-of-care applications, compact NIR and Raman systems have become essential. This section examines how advances in micro-optics, MEMS technology, and fibre-optic probe design have enabled the development of portable, battery-powered devices that can deliver laboratory-grade information outside of controlled environments. We also present representative examples of how these compact systems are being used for food safety and clinical monitoring.

### 2.1. Evolution of Miniaturised NIR Spectrometers

Miniaturised devices have external radiation sources and are mains-powered. They have a limited wavelength range and low resolution, and the spectra are not reproducible. The optical window is also limited in size. It is also necessary to connect to an external computer, so they cannot be used in the field. With advancements in technology, medium-sized sensors that are handheld systems have been developed. These handheld systems include all the necessary components to be used autonomously, such as battery and electronic control systems. The most representative and widely-used instrument in miniaturised NIRS is the Phazir™ NIR Instrument (currently distributed by Thermo Fisher Scientific, 3rd Avenue, Waltham, MA, USA). The rapid development of microelectronics as well as micro-electro-mechanical systems (MEMS) has allowed the transition from medium-sized equipment (around 1.5 kg) to microspectrometers (over 100 g) in the last few years. In this case, the device used most is the MicroNIR 1700 (VIAVI Solutions Inc., Chandler, AZ, USA).

**Figure 1:**
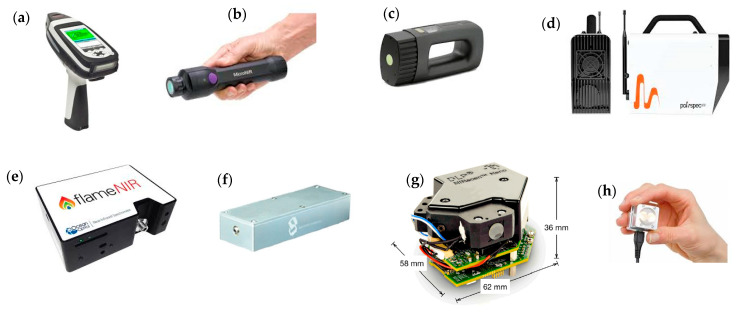
Most common handhelds near-infrared spectrometers: (**a**) MicroPHAZIR™ (Thermo Fisher Scientific); (**b**) MicroNIR Pro ES 1700 (VIAVI); (**c**) NeoSpectra (Si-Ware Systems, Menlo Park, CA); (**d**) Polispec NIR (ITPhotonics); (**e**) Flame-NIR (Ocean Optics); (**f**) nano-FTIR NIR (Southwest Technologies Inc., Menlo Park, CA, USA); (**g**) DLP^®^ NIRscan™ Nano EVM (Texas Instruments); (**h**) NIRONE Sensor S (Spectral Engines, Steinbach, Germany).

Beć et al. [1] examined the fundamental principles and diverse applications of miniaturised near-infrared (NIR) spectrometers. Of particular note is the broad and diverse range of applications, which span agriculture and the food industry, materials science, industrial processes, and environmental monitoring. Unlike the relatively uniform and mature design of benchtop FT-NIR spectrometers, miniaturised instruments incorporate a variety of technological solutions that directly impact their operational performance. Steady technological progress has led to a continuous stream of new devices entering the market. This has shifted the current focus in analytical NIR spectroscopy towards systematically evaluating these instruments, refining associated methodologies, and comprehensively characterising their performance profiles. Figure 1 shows the eight most common miniaturised NIR instruments. The characteristics of these NIR miniaturised instruments are listed in Table 2.

### 2.2. MEMS-Based Architectures and Smartphone Integration

Microelectronics and MEMS or microfabrication were used in some portable instrument manufacturers to produce tiny devices which were then assembled into complete instruments. These instruments are battery powered, have internal radiation sources (intended for reflectance measurements), and offer modern wireless digital data exchange technologies, such as Bluetooth^®^. They operate in a stand-alone mode and are referred to as “micro” instruments due their low weight (about 100 g) and compact size, approximating in average a 10 × 10 × 10 cm^3^ or smaller box.

The specifications listed in Table 2 directly affect the analytical performance of miniaturised NIR instruments. The spectral range determines which chemical bonds can be probed. Devices operating only in the 900–1700 nm region are well-suited to estimating moisture, proteins, and fats, whereas those operating over a broader range (up to 2500 nm, for example) capture stronger combination bands, which improves sensitivity for complex matrices. The spectral resolution of an instrument influences its ability to distinguish between overlapping absorption features. Higher resolution is particularly important when analysing samples with subtle compositional differences or quantifying minor constituents. In practice, handheld devices with moderate resolution are capable of robust screening tasks, but high-resolution instruments are still advantageous for demanding quantitative applications. Finally, the type of detector, the optical design, and the illumination geometry affect the signal-to-noise ratio and the measurement stability. These factors are critical for on-site use, where environmental conditions are less controlled. Together, these factors determine whether a device is suitable for rapid screening, in-line monitoring, or more rigorous, laboratory-grade quantification.

**Figure 2:**
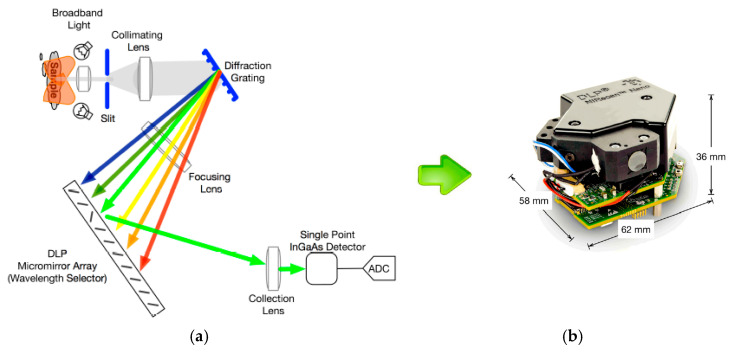
(**a**) Texas Instruments DLP-based architecture; (**b**) DLP^®^ NIRscan™ Nano Spectrometer dimensions [5].

A notable example of miniaturised NIR devices is the DLP^®^ NIRscan™ Nano Spectrometer [4]. This device is based on a digital micromirror device (DMD) designed by Texas Instruments, combining a light detector and a grating. It uses digital light processing (DLP) technology according to the principle of a monochromator. Figure 2 illustrates the architecture of the spectrometer based on DLP technology. The key component is the DMD microarray. This consists of a matrix of thousands of micromirrors, each of which has a surface angle that can be controlled by applying a voltage. Individual wavelengths are selected by turning columns of mirrors on or off to reflect only the desired wavelengths to the detector. This technology enables a larger detector area and the use of a single light detector.

While developing compact instruments is one of the main research lines, integrating microspectrometers into smartphones is one of the latest and most common trends. In [5], Huang et al. described the development of smartphone-based NIR fluorescent imaging technology and the quality and potential of point-of-care applications. Another key goal for the future is the promotion and application of cloud computing platforms (IoT technology); however, despite the development of a large number of new chemometric methods, instrument software is often not updated for the development of these methods. Wang et al. reviewed various chemometric methods that have been applied in modern spectral analysis over the past ten years, particularly from a practical perspective [6].

With cloud platforms, spectra data from different sources, such as different points on the production line, can be managed and stored, and can enable automatic optimisation processing and big data analysis [7,8]. Even with all the benefits of miniaturised devices, sampling can still be highly difficult in certain circumstances.

### 2.3. Fibre-Optic Probes for In Situ and Non-Invasive Measurements

One of the best instrumental solutions is to employ fibre optics, either single or multiplexing fibres, for parallel processing. Fibre-optic probes have emerged as indispensable tools in modern spectroscopy, renowned for their versatility across the visible and near-infrared frequency ranges [3]. Fibre-optic probes typically consist of separate illumination and collection optical fibres connected to the light source and detector, respectively. They are bonded to the probe tip to allow for simultaneous illumination of the sample and collection of the light. The geometry of the probe tip varies depending on the optimal coupling to the sample or the geometry of the light emission from the probe tip.

Optical fibres enable the transmission of light to and from biological tissues, achieving the measurement of various physiological parameters without the need for invasive procedures. NIRS, for example, can be employed to analyse the chemical composition of tissues and body fluids, providing valuable insights into blood oxygenation, tissue perfusion, and metabolic activity [9]. When contrasting miniaturised equipment with fibre-optic probes and laboratory instruments, it has been observed that, despite the lower performance, the ability to conduct on-site and in-line analyses is of greater interest to the industry than the potential decrease in accuracy [10]. The employment of optical fibres for non-invasive clinical analysis enhances patient comfort, reduces the risk of complications, and improves the efficiency and accuracy of medical diagnostics.

**Figure 3:**
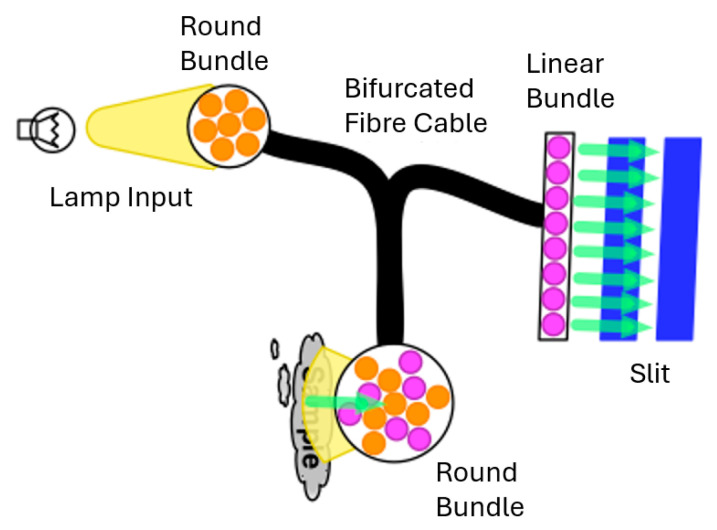
Configuration of a fibre-optic bundle reflectance probe.

The most basic type of fibre-optic probe is a reflectance probe. The fibre bundle is usually divided into two parts (see Figure 3): one set of fibres is routed to a light source, while the other set of fibres captures the reflected light and directs it to the input slit. DLP technology-based spectrometers have tall slits; thus, when combined with round-to-linear fibre bundles that fill the complete slit, the highest performance is obtained. Before reflection data can be collected by the spectrometer, the system must be calibrated by taking a reference scan. To take this reference scan, diffuse reflectance material, such as Spectralon, is placed in the same position relative to the probe as will be used for the actual measurement.

### 2.4. Practical Applications in Food Safety and Clinical Monitoring

The rapid development of handheld and micro-NIR spectrometers has had a direct impact on food safety and clinical applications. In the food industry, for example, portable NIR devices are now routinely used to quickly screen for moisture, protein, fat, and adulterants in grains, dairy products, meat, and oils. This enables quality control at reception points and along production lines. In health-related contexts, fibre-optic NIR probes allow non-invasive monitoring of tissue oxygenation, perfusion, and metabolic status, supporting point of care diagnostics and continuous physiological assessment. These examples illustrate how the instrumental trends described above—miniaturisation, MEMS-based architecture, and improved probe designs—are directly enabling real-time decision-making in both food safety and clinical environments.

## 3. Advances in Raman Technologies and SERS Substrates

The second dominant trend is the expansion of Raman-based technologies, particularly those designed to overcome the inherently weak Raman signal and fluorescence interference. Recent developments, such as surface-enhanced Raman spectroscopy (SERS), surface-enhanced Raman scattering (SORS), and Raman hyperspectral imaging, have significantly enhanced sensitivity and depth-profiling capabilities. Meanwhile, portable and handheld Raman instruments have become more robust and accessible. This section reviews these technological advances and demonstrates how they enable the practical, on-site detection of contaminants, adulterants, and clinically relevant biomarkers.

While the NIR spectrum is due to radiation absorption, the Raman spectrum is related to scattering effects. This type of spectroscopy uses a chromatic laser to excite molecules, and the Raman spectrum is observed when they return to their initial vibrational level through inelastic scattering. For highly hydrated samples, Raman spectroscopy has several advantages over NIRS, including minimal scattering of the polar O-H group and more intense bands of homonuclear groups. However, one of the drawbacks of Raman spectroscopy is the low fraction of scattered photons (often lower than 0.0001%), which results in low signal-to-noise ratios and long acquisition times.

Raman techniques enable signals to be collected from food and clinical samples. In addition to standard spectral collection, advanced Raman technologies, such as surface-enhanced Raman scattering (SERS), spatially offset Raman spectroscopy (SORS), and the emerging Raman hyperspectral imaging (RHSI), are now the most widely employed. In [11], Sun et al. presented the advantages of Raman technologies and applications in the food-production chain. SERS has emerged as an alternative to the low cross-section of Raman scattering (10^−28^ cm^2^ per molecule). It includes metallic nanostructures near or absorbed by the target analyte to enhance the Raman scattering signal. SORS is a technology that spatially separates the excitation laser and the signal detection, enabling it to probe signals from deeper layers of a sample. An alternative way to improve and increase the SORS spectra is to use hyperspectral Raman imaging, which allows the spectral and spatial resolution to be obtained simultaneously. All of these Raman techniques can be used for qualitative and quantitative analysis.

One area of Raman analysis is the design of miniaturised devices to be used as detectors in different miniaturised analytical techniques, such as lab-on-a-chip (LOC) [12], microfluidic [13], and lateral flow [14]. These analytical strategies often use nanomaterials to enhance the Raman signal (SERS), and have all been developed as on-site sensor procedures for detecting target analytes in specific samples. They frequently require sample pre-treatment to extract the analyte prior to analysis, and this process is usually straightforward. LOC is an emerging strategy because multiple components (i.e., sample extraction, preconcentration, and detection) are integrated into a chip. The Raman signal can be used as a final signal in (bio)chemical sensors or a technique to detect unknown molecules or to quantify specific compounds by combining spectra and chemometrics.

Similarly to NIRS, Raman techniques can be used to scan samples on-site with portable, handheld instruments or with a modular system. Raman miniaturisation is not limited to reducing physical dimensions but must also ensure adequate performance for specific applications. Today, handheld Raman devices are used across diverse fields, including food safety, where they enable the rapid detection of adulterants and counterfeit products, and medicine, where their integration with wearable SERS sensors supports non-invasive biomolecule detection and potential early disease diagnosis. Handheld Raman technologies have also expanded into dermatology, allowing for in-skin compound analysis through confocal probes, and into public safety, where remote handheld systems can identify hazardous substances with precision comparable to benchtop instruments. Recently, handheld FT-Raman spectrometers have been deployed for on-site drug analysis. A growing trend is the integration of Raman spectroscopy with smartphones, either through embedded modules or detachable designs, improving portability and usability. When combined with SERS substrates and cloud-based processing, smartphone-based Raman systems can leverage deep learning to enhance spectral interpretation, mixture discrimination, and quantitative accuracy [15].

With portable Raman spectrometers (sometimes called compact spectrometers), the user can streamline and customise a Raman system to fit their specific application, reducing size, weight, complexity, and cost in the process. The small size and robustness of these devices make them easy to move from one location to another, yet they are powerful enough to carry out analytical measurements on-site.

**Figure 4:**
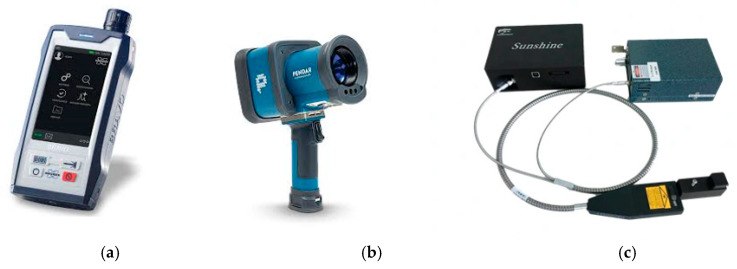
Raman Instrumentation: (**a**) Handheld Raman spectrometer Bravo (Bruker); (**b**) Handheld Raman spectrometer Pendar X10 (Pendar Technologies); (**c**) Raman modular system (CNI).

Handheld Raman instruments (see Figure 4a,b) are designed for use in the field by non-experts, providing definitive identification of a substance with a simple point-and-click operation. They integrate a single-wavelength Raman system, sampling optics, data processing, and a display screen/user interface into a lightweight, battery-powered unit robust enough for use by first responders and in manufacturing environments. They deliver rapid and reliable results but are limited to the installed libraries. A third option is the Raman modular system. This system consists of a spectrometer, an external laser, flexible fibres, and a Raman probe.

The parameters summarised in Table 3 also have significant practical implications for Raman performance. The choice of laser wavelength is particularly important. Shorter wavelengths (e.g., 532 nm) generate stronger Raman scattering, but they also induce higher fluorescence, which can obscure weak signals in biological or food samples. Although longer wavelengths (e.g., 785 nm or 1064 nm) reduce fluorescence and improve selectivity for complex matrices, they also result in lower scattering efficiency. The sensitivity and penetration depth of a laser are influenced by its power. However, excessive power can cause sample heating or photodegradation, particularly in biological tissues. The ability to resolve the narrow Raman bands associated with specific molecular vibrations is determined by spectral range and resolution. High resolution is essential for discriminating between structurally similar compounds or for detecting trace contaminants. In real-world applications, these trade-offs influence the selection of instruments: Handheld systems operating at 785 nm are typically preferred for food authentication and contaminant screening, while systems operating at 1064 nm are advantageous for highly fluorescent clinical samples.

This allows us to build a ‘tailor-made’ system by choosing the laser wavelength, probe type, spectral range, resolution, and detector type, and this gives researchers, laboratories, and industries great flexibility. Figure 4c shows a low-cost modular system from CNI, and Table 3 provides the specifications of some popular Raman spectrometers.

The evolution of portable Raman and SERS systems has also resulted in significant progress in health and food safety control. Handheld Raman instruments are increasingly being used to rapidly identify food adulterants (e.g., melamine, illegal dyes, and pharmaceutical residues), to authenticate high-value products, and to detect spoilage markers. In clinical and biomedical settings, sensors based on SERS integrated into microfluidic or wearable platforms can detect biomarkers such as glucose, lactate, urea, and inflammatory molecules at trace levels. These practical applications demonstrate how improvements in excitation sources, detector sensitivity, and nanostructured substrates can support the deployment of Raman technologies in real-world safety and diagnostic workflows.

It is important to note that Raman scattering and fluorescence often coexist as competing effects, making it challenging to distinguish weak Raman signals from the fluorescence background. This necessitates the development of instrumentation capable of distinguishing between Raman and fluorescence events. The selection of the excitation laser and imaging spectrometer is critical to the success of the final application.

## 4. Hyperspectral Imaging (HSI) and Deep Learning Approaches

The third trend driving innovation in vibrational spectroscopy is the integration of hyperspectral imaging (HSI) with advanced computational methods. HSI combines spectroscopy and imaging to provide spatially resolved chemical information, which is particularly valuable for heterogeneous food matrices and biomedical tissues. Recent advances in deep learning have further improved the extraction of spectral–spatial features, allowing for more precise classification and quantification. This section outlines the principles of hyperspectral imaging (HSI), emerging Raman and near-infrared (NIR) imaging platforms, and the growing role of deep learning in processing high-dimensional spectral data.

HSI uses a combination of imaging and spectroscopy to collect spatial and spectral information from materials simultaneously. This enables a detailed chemical and structural analysis. Although HSI was initially developed for remote sensing, it is now increasingly being used in biomedical diagnostics, clinical surgery, pharmaceutical inspection, agricultural monitoring, and food safety control. This technology is highly effective in detecting subtle biochemical differences, measuring physical properties, and identifying contaminants without destroying the sample. Pallua et al. [16] presented new applications of hyperspectral imaging in clinical research.

HSI systems generate rich datasets known as ‘hypercubes’, comprising two spatial dimensions and one spectral dimension. Each pixel contains a full spectrum of reflectance, absorbance, or fluorescence, enabling researchers and practitioners to accurately classify materials, to measure chemical concentrations, to detect diseases, and to identify contaminants. HSI has quickly started using real-world health and food safety applications, having initially started with laboratory demonstrations. The most significant trends are as follows: (1) the use of deep learning and transformer-style models for tasks involving both spectrum and space; (2) miniaturisation, with the development of portable and handheld devices enabling on-site screening; (3) a push towards in-line/real-time HSI on production lines, via push-broom and snapshot designs, as well as edge inference; (4) increased interest in short-wavelength infrared (SWIR) and multimodal fusion, when chemical or penetrative information is required; (5) a focus on domain shift, few-shot learning, transfer learning, and explainability. These approaches are being developed to address the variability across different sites and seasons. Nevertheless, cost, standardisation, data volume, calibration/illumination dependency, and regulatory validation continue to be the main barriers to the widespread adoption of this technology in industry and clinical practice. Kumar et al. provided a thorough survey of DL for HSI classification [17].

Despite these advances, there are still several fundamental barriers that limit the large-scale deployment of vibrational spectroscopy and HSI systems in operational environments. Calibration and calibration transfer remain among the most persistent challenges. Models developed on one instrument or in one location often do not work when applied to different devices, illumination conditions, or sample populations. A domain shift, which can be caused by seasonal variability, biological diversity, changes in production, or sensor drift, can significantly reduce the robustness of a model, particularly for machine learning-based approaches. Miniaturised NIR and Raman devices are becoming more affordable, but high-performance HSI systems, SERS substrates, and specialised optics still require substantial investments and technical expertise. Together, these factors slow down the industrial and clinical adoption of technology, highlighting the need for standardisation, adaptive modelling strategies, and cost-effective hardware solutions.

Modern systems typically employ convolutional neural networks, spectral–spatial architecture and, increasingly, transformer-based models to extract subtle features from cubes for classification, segmentation, and regression. Although these models significantly improve detection and quantification performance compared to classical chemometrics (PLS and SVM), they require larger labelled datasets and more computing power. Recent surveys have suggested that transformer and contrastive transfer approaches are increasingly popular ways of addressing spectral complexity and domain shifts. On the other hand, several studies and reviews have demonstrated the effectiveness of portable HSI technology for on-site meat authentication, for screening snacks and produce, and for testing grain quality. These studies often use push-scan sensors and lightweight embedded processors. This makes it possible to carry out field and plant inspections, as well as in situ factory checks, for which laboratory spectroscopy was previously required. Bruno et al. demonstrated that portable and handheld HSI systems can be used as a rapid, non-destructive technique to predict the acrylamide content of potato crisps [18].

Push-broom line-scan setups remain dominant for high throughput. However, snapshot imagers combined with optimised inference (quantised models, GPU/FPGA/edge inference) are enabling real-time foreign object detection and composition mapping (fat/moisture), and sorting on conveyor belts. Several groups have reported improvements in production rates in agriculture and food processing by using AI-assisted push-broom systems. Neri et al. presented the development and implementation of a real-time, AI-assisted, push-broom, hyperspectral system for identifying plants [19].

When surface colour alone is insufficient for identification purposes (e.g., for detecting internal defects, polymers or chemicals, or mycotoxin proxies), SWIR (1000–2500 nm) provides more direct compositional contrast. Snapshot spectral imagers are ideal for applications where motion or speed is critical. Recent reviews have emphasised the importance of matching the spectral range and sensor architecture to the chemical target. Thomas et al. identified several trends in snapshot spectral imaging [20]. Combining HSI with RGB, thermal, Raman, fluorescence, or chemical/targeted sensors can make it possible to distinguish between cases where spectral signatures are difficult to separate (e.g., microbial and chemical contaminants, or visually similar adulterants). Fusion strategies, such as early and late fusion, as well as learned fusion, are a current area of research. Medina-García et al. explored strategies for analysing hyperspectral imaging (HSI) data to identify issues relating to the quality and safety of food [21].

Real-world deployments have demonstrated that models trained in one season, factory, or instrument tend to perform poorly in others. Recent work has focused on transfer learning, contrastive and few-shot methods, domain adaptation, and instrument standardisation procedures. The aim is to reduce the need for relabelling. Cross-domain hyperspectral imaging (HSI) classification and few-shot learning are active and practically relevant topics. Jiang et al. discussed the task of cross-domain HSI classification in great detail. [22]. However, high-performance HSI cameras with a broad SWIR range and high spectral resolution remain more expensive than RGB/mono cameras. The cost is increased by the need for specialised optics, stable illumination systems, and robust housings, particularly for SWIR and cooled detectors. Cost is repeatedly cited as the main barrier to adoption in reviews and application papers. In [23], Li et al. presented the latest advances and challenges in the use of hyperspectral imaging (HSI) to detect foreign matter in food products and to outline future directions in this field.

HSI cubes contain hundreds of bands and have a high spatial resolution, meaning they are orders of magnitude larger than RGB frames. This creates bottlenecks in terms of storage, transfer, and computing, particularly for long-term production logging and traceability. Heavy preprocessing is required, including calibration, dark/current correction, and reflectance conversion. HSI-derived features are sensitive to the spectrum, angle, and environmental conditions of the illumination. Without strict spectral and radiometric calibration and controlled lighting, the accuracy of the models deteriorates rapidly. Many practical deployments fail to allocate sufficient funds for regular spectral reference checks and maintenance. Spectral signatures may differ due to variations in instrumentation, ageing, surface wetness, cultivar/variety, and seasonal changes. Cross-domain failure is well documented. While model transfer methods are necessary, they are not infallible. Building robust classifiers often requires chemically verified ground truth, such as laboratory assays, microbiology, and GC/MS analysis of contaminants. However, these assays are expensive and time-consuming, which limits the amount of labelled data available and slows down model development. Several food safety reviews have highlighted the lack of labels as a bottleneck. Guo et al. conducted a thorough investigation into the use of HSI technologies for detecting fungal and mycotoxin contamination in grains and oilseeds [24].

Although clinical HSI research is promising, regulatory approvals and large prospective trials remain scarce. Standardised clinical endpoints, safety profiles, and acceptance criteria are under development. Standardised test protocols for food safety are still being developed across vendors. In their review, Ali et al. demonstrated the significant potential of HSI as an intraoperative guidance tool for assisting surgeons during tumour resection by generating detailed tissue density maps [25]. Even a low rate of false positives or false negatives can result in significant operational costs in production lines (e.g., wasted product, rework, line stoppage). Integrating HSI requires production engineers to co-design optics, mechanical positioning, preprocessing, and AI thresholds [19].

## 5. Chemometrics and Machine Learning for Robust Modelling

The fourth major trend is an increasing reliance on chemometrics and machine learning for interpreting complex vibrational spectra. As portable instruments generate larger and more varied datasets, robust modelling strategies are essential for reliable decision-making. This section reviews classical chemometric tools and modern machine learning approaches, highlighting their respective strengths and limitations, as well as their suitability for real-time applications. This section provides an overview of classical chemometric tools and modern machine learning approaches. It highlights the strengths and limitations of each approach and considers their suitability for real-time applications.

**Figure 5:**
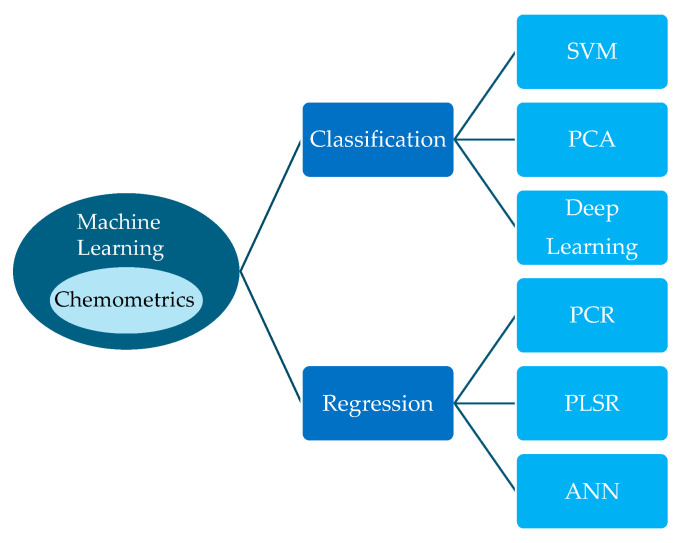
Techniques for classification and regression of health and food safety control (SVM = support vector machines, PCA = principal component analysis, PCR = principal component regression, PLSR = partial least squares regression, ANN = artificial neural network).

Vibrational spectroscopy generates huge amounts of spectral data. Processing this data is a critical step involving preprocessing, calibration, and analysis. Advanced algorithms, including chemometrics and machine learning, are used to extract such meaningful information (qualitative and/or quantitative) as nutrients, moisture, and disease. Chemometrics involves analysing chemical data using specialised statistical tools. Machine learning, on the other hand, is a general-purpose pattern-learning framework that is used across many domains, including chemistry. Figure 5 shows some of the classification and regression techniques that are used in food safety and health control.

The accuracy and reliability of the information derived from the spectral data is increased through this processing. Integrating this technology into the agri-food and medical sectors requires developing accurate models with high inter-instrument reproducibility, which currently necessitates investment in the development and maintenance over time of robust chemometric models. Nowadays, to develop and maintain calibration models requires specialised personnel, which significantly limits their practical utility. For this reason, one of the future research trends is focused on the development of new algorithms addressed to reduce the workload associated with model establishment and maintenance.

As previously mentioned, vibrational spectroscopy generates a large-scale database. Typically, in common supervised learning strategies, each spectrum is labelled with reference data (e.g., parameter concentration or sample type). However, one of the main emerging trends from the large amount of vibrational spectroscopy data is the development of semi- or unsupervised models that can extract relevant information for future applications [6].

One of the most remarkable aspects of the current trends is that it is becoming more and more common to use two or more techniques in order to obtain the maximum information. This means that classical chemometric methods, such as principal component analysis (PCA) and partial least squares regression (PLSR), sometimes fail to produce satisfactory models. In recent years, multi-block methods have been developed to perform these analyses. Multi-block analysis enables complementary information to be obtained from different techniques. Mishra et al. provided an overview of the multi-block data analysis concept in [26], while Smilde et al. provided a framework for this subfield of data fusion [27]. This type of analysis identifies common and distinct information from data obtained from different sensors. In [28], Song et al. proposed a model for addressing the issue of mixed data types in multiple datasets. It is important to note that machine learning algorithms are being applied in the development of new and robust calibration models. The most actual deep learning approaches, especially convolutional neural networks (CNN), have started to be applied because neural networks are not required to select variables before model development. They use multiple convolution layers and pooling layers to extract the relevant information. Zhang et al. discussed the challenges currently associated with conventional chemometric methods and deep learning (DL) to spectral analysis [29].

To add to the development of robust calibrations, it would be a challenge for the development of learning strategies to be able to offer the possibility of transferring calibrations from one instrument to another, moving vibrational calibration models to a universality, not depending on the instrument employed to analyse the sample. According to Mishra et al. in [30], the translation of NIR technology into commercial slaughterhouse conditions is challenging. Yang et al. demonstrated that combining HSI and DL increases the accuracy and efficiency of inspections of fruit and vegetables [31]. The future research should, however, emphasise cost reduction, equipment optimisation, feature extraction personalisation, and model generalisability.

The adoption of ML models in the industrial sector for food safety purposes is still in its early stages. This is because there are some issues with how these models are designed and how they are used in practice. PLS-based models are reliable, but their robustness for industrial deployment is lacking. The methods for analysing samples and categorising them also need to be improved. HSI was adopted to evaluate the ripeness degree of kiwifruit [32]. To address these issues, there is a pressing need for industrial-focused research on ML model adaptations, particularly models for staple foods which are integral to food security. Implementing kernel functions within these models and focusing on more specialised, food-specific algorithms would bridge the existing research gaps and make ML models more applicable to industrial food safety contexts.

The selection of PCA, PLS, or deep learning models for NIR and Raman spectroscopy depends strongly on the analytical context, especially in regulatory and industrial environments where robustness, interpretability, and model transferability are essential. PCA and PLS are chemometric tools easy to understand, and they perform well even with small or moderately-sized datasets. They are both preferred tools for exploratory analysis, quantitative modelling, and quality control applications. By contrast, while deep learning approaches are powerful for handling large spectral datasets and for capturing non-linear relationships, they still face challenges in terms of interpretability and standardisation, which limits their regulatory acceptance despite their growing relevance in biomedical and food analysis scenarios [33,34].

Looking to the future, chemometrics and machine learning offer clear benefits for food safety tasks, such as faster pathogen detection, predictive outbreak forecasting, automated visual inspection, and richer supply chain traceability. However, progress is hindered by inconsistent data, poor model interpretability and validation, limitations in sensors and integration, and regulatory and operational barriers. In order to progress from promising pilots to reliable, trusted systems, we require standards for data and evaluation, privacy-preserving distributed learning, and multimodal models that are linked to domain knowledge. Furthermore, regulatory pathways are needed for the deployment of these systems to be validated. Zhang et al. reviewed the transformative role of ML and DL in enabling intelligent food safety management through the efficient analysis of high-quality, non-linear data [35].

### Chemometrics Software Tools

Chemometrics has become an essential component of modern analytical science. It enables meaningful information to be extracted from complex datasets generated by spectroscopy, chromatography, hyperspectral imaging, and biosensing platforms. While historically rooted in multivariate statistics, the field has evolved to encompass advanced machine learning and data-driven modelling techniques. As industries adopt increasingly automated, data-intensive workflows, chemometrics software tools are undergoing a significant transformation.

The global chemometrics software market is experiencing sustained growth, driven by the increasing availability of high-dimensional analytical data and the requirement for robust and interpretable models in regulated environments [36]. The key drivers are as follows:

- The expansion of process analytical technology (PAT) in pharmaceutical manufacturing.
- Increased adoption of hyperspectral imaging for food quality control and agricultural monitoring.
- The emergence of cloud-based infrastructure that supports scalable computation and collaborative workflows.
- Regulatory pressure for traceable, auditable, and validated analytical models.

These factors collectively position chemometrics as a strategic capability across multiple industrial sectors.

Despite the rise of machine learning, classical chemometric methods remain fundamental. Principal component analysis (PCA), partial least squares (PLS) regression, discriminant analysis, and standardised preprocessing techniques (e.g., SNV, MSC, and their derivatives) continue to form the core of most software platforms. Recent developments have focused on improving usability, automating preprocessing pipelines, and enhancing visualisation capabilities.

A defining trend is the integration of machine learning algorithms into chemometric workflows. These tools are increasingly incorporating the following: support vector machines, random forests, gradient boosting methods, neural networks, and deep learning architecture.

The aim of these hybrid approaches is to combine the interpretability of classical chemometrics with the predictive power of modern machine learning, particularly in applications involving non-linear relationships or large-scale imaging datasets.

On the other hand, cloud computing is transforming the field of chemometrics. Cloud-native platforms offer elastic computing resources for large-scale datasets, centralised model management, and seamless collaboration across a geographically distributed team and continuous updates without local installation constraints. This shift is particularly impactful for hyperspectral imaging and omics-related applications, where substantial data volumes are involved.

Industrial environments demand real-time decision-making. Emerging software solutions support instrument chemometric modelling, edge computing for embedded systems, and continuous monitoring and adaptive control. These capabilities are essential for the pharmaceutical industry, for food processing, and for environmental monitoring. Chemometrics is increasingly used for authenticity testing, fraud detection, freshness assessment, and shelf-life prediction. Integration with hyperspectral imaging and machine learning is accelerating adoption. Remote sensing and drone-based hyperspectral imaging are expanding the use of chemometrics in crop monitoring, soil analysis, and precision agriculture. Multivariate analysis supports biomarker discovery, omics integration, and interpretation of complex biosensor signals. Chemometrics is increasingly embedded in point of care diagnostic systems.

The number of chemometrics software tools available for developing the trained model is just as numerous as the mathematical methods. Depending on the needs (spectroscopy analysis, calibration, data exploration, regression, classification, etc.), there are several widely used chemometrics tools, both commercial and open source. Table 4 shows some of the most popular and well-established chemometrics software packages. These tools continue to evolve while new entrants introduce cloud-native, machine learning-centred, and domain-specific solutions. User experience, automation, and interoperability are becoming major differentiators.

The future of chemometrics software will be shaped by several converging trends:

- Hybrid modelling frameworks combining classical chemometrics with advanced machine learning.
- Explainable AI to ensure transparency in complex models.
- Scalable cloud infrastructures for large-scale imaging and omics data.
- Regulatory-ready digital ecosystems supporting model lifecycle management.
- Integration with microfluidics and biosensing platforms for real-time analytics.

In conclusion, chemometric software tools are undergoing a significant transformation driven by technological innovation, regulatory demands, and the growing complexity of analytical data. The integration of cloud computing, machine learning, and automated validation is reshaping how chemometric models are developed, deployed, and maintained. As industries move toward real-time, data-driven decision-making, chemometrics will continue to evolve into a foundational component of modern analytical science.

## 6. Applications in Food Authentication and Health Control

Miniaturised and low cost NIR sensors have been widely employed for quality and safety controls in a wide variability of foods, such as vegetables, meat, milk, fish, among others [37,38,39,40,41]. The most recent applications combine portable instrumentation and chemometrics to extract relevant and safe information and to mark the importance of optimising the new generation of NIR instruments for on-site and in-line NIRS analysis.

### 6.1. Applications in Food Authentication

Microbiological control is of paramount importance in ensuring food safety. Bacteria, viruses, parasites, and fungi can contaminate food at any stage of production, posing serious health risks to consumers if ingested. Through rigorous microbiological testing and monitoring, food producers can identify and mitigate potential hazards, ensuring that food products are safe for consumption. In essence, microbiological control plays a crucial role in preventing foodborne diseases and protecting the well-being of individuals and communities. However, non-invasive and real-time analysis strategies need to be developed to increase sampling and to improve food safety tools.

Vibrational spectroscopy is a good alternative to classical methods of identification and control of microbiological growth, which are usually expensive, require skilled personnel, and are difficult or not possible to carry out in situ. In their literature review, Tian et al. [42] described the different ways of applying near-infrared spectroscopy for microbiological control, including examples of the identification and classification of pathogenic bacteria in foodstuffs as well as monitoring systems. HSI combined with machine learning has allowed us to obtain a lot of information about products with complex matrices such as vegetables and cereals [43].

One of the advantages of the HSI technique is its capability to provide both spectral and spatial information. This feature enables the analysis of complex and non-homogeneous samples. For instance, in a study by Hardy et al. [44], salmon fillets were examined using absorption spectroscopy with a hyperspectral camera over four storage days. Classification methods such as K-nearest neighbors (K-NN) and PCA were utilised, achieving accuracy of 77.0% and 73.7%, respectively, in predicting the storage day. The use of hyperspectral imaging unveiled increased spoilage in the tail bottom fillet sections as the fillets aged, with the dampening of an absorbance band around 600 nm identified as the primary indicator distinguishing between fresh and spoiled samples. This highlights the significance of considering spatial inhomogeneity in studies on fish freshness, with HSI proving to be a valuable tool for such analyses.

In addition to microbiological contamination and the sale of adulterated or non-fresh foods, the presence of microplastics in food poses a significant threat to food safety. A study by Aramendia et al. adopted an analytical approach to confirm the internalisation of microplastics in cryogenic cross-sections of muscle tissue. Utilising 3D Raman confocal microscopy with chemometrics, microplastics measuring 1 μm were successfully identified. Optical and fluorescence microscopy further validated the findings. The investigation unveiled evidence of microplastic internalisation in the digestive epithelial tissues of exposed mussels (*Mytilus galloprovincialis*) [45].

Raman is also a very useful alternative for food safety. It can be a tool to guarantee food safety for inspection services or consumers. It has been used to detect fungi, toxins, and frauds, among others. Raman spectra combined with proper chemometrics provides a fingerprint detection of mycotoxins and, when using specific antibodies or aptamers, the selective quantification can be carried out by SERS. Future work should be developed to implement SERS detection in smartphones integrating the spectrum collection, processing, and results [46]. Using a handheld Raman spectrometer and partial least squares discriminant analysis (PLS-DA), Logan et al. [47] demonstrated the viability of the Raman spectrum to correctly classify Australian beef; differences in spectra were observed predominantly at 1301, 1440, and 1658 cm^−1^.

### 6.2. Applications in Health Control

Concerning the use of these techniques in the field of health, one of the most established uses of vibrational spectroscopy is in the area of neurology. The central nervous system is difficult to access and, in many cases, information is required from complex areas in which invasive interventions would not be possible. In such situations, non-invasive assays can be used for functional near-infrared spectroscopy (fNIRS), and a non-invasive neuroimaging method can be useful to study cortical activity by tracking hemodynamic responses during human movement. Its portability and low sensitivity to motion artifacts have made it widely used for assessing cortical responses in motor tasks [48]. Khan, M.A. et al. [49] investigate ways to improve the accuracy of cognitive workload detection by combining fNIRS signals with advanced deep-learning models.

As mentioned previously, one of the qualities that make these non-invasive and non-destructive techniques so interesting is that they allow for an ex vivo analysis. Yim et al. [50] assessed how well near-infrared spectroscopy (NIR) can distinguish between benign and malignant bladder conditions right after surgery, considering the severity and stage of cancer. They analysed 355 spectra from 71 bladder samples taken during tumour removal surgeries. Using a portable NIR device, they scanned the samples within 10 min of removal. After a routine lab analysis, machine learning models were applied to develop diagnostic tools based on the spectra data. The results showed high accuracy in identifying low- and high-grade cancers (97% sensitivity, 99% specificity) and for predicting different cancer stages (97% sensitivity, 92% specificity). This suggests NIRS’s potential for the quick assessment of bladder tissues post-surgery, which could help guide treatment decisions and reduce the need for further procedures [50]. The authors agreed that further research with fibre-optic probes may enhance real-time tissue evaluation.

**Figure 6:**
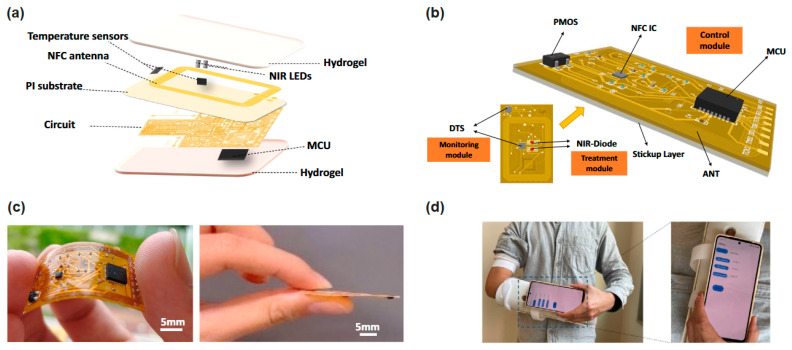
e-Bandage structure for cutaneous bounds: (**a**) e-Bandage structure; (**b**) Surface-mount components; (**c**) Flexible e-Bandage circuit before encapsulation; (**d**) Stable wireless monitoring for wounds protected by plaster [51].

Vibrational radiation has also demonstrated excellent potential in the therapeutic field. In recent research work, Yu et al. [51] described an innovative application to treat wounds and other cutaneous diseases, using an e-bandage device by combining smartphone and NIRS. The energy collected using NIR light emitting diodes (LEDs) with a central wavelength at about 940 nm and temperature sensors that monitor the healing process by comparing temperature in the middle of the wound and in healthy regions become the e-banding in this system. Figure 6 shows the structure of the e-bandage developed for the cutaneous bound treatment.

Portable Raman devices have also been investigated as an alternative for the in vivo diagnosis of skin cancer. A large database including 615 patients and PLS-DA as chemometric tool were employed to correctly classify in vivo Raman spectra. Prior to scanning skin in this assay, a specialised oncologist examined the patients and, after selecting the damaged area, proposed the scanning zone of the tissue skin collection spectra [52].

## 7. Calibration Transfer, Validation, and Regulatory Readiness

The final trend identified in this review is the increasing focus on calibration transfer, validation protocols, and regulatory compliance. As vibrational spectroscopy moves from laboratory research to industrial and clinical practice, it becomes critical to ensure method robustness across instruments, environments, and sample types. This section examines the strategies for calibrating benchtop and portable devices, discusses the validation requirements for on-site deployment, and outlines the regulatory considerations relevant to food safety and medical diagnostics. These aspects are essential for achieving reliable spectroscopic workflows that can be deployed in the field.

The consolidation of vibrational spectroscopy as a reliable tool for food safety and health applications increasingly depends on its ability to deliver reproducible, auditable, and regulatory compliant results. In this context, calibration transfer, robust validation, and alignment with regulatory frameworks have become key trends in the real-world deployment of these technologies outside of the laboratory [10].

One of the main challenges in the practical implementation of spectral models is their dependence on the instrument and acquisition conditions. Models developed using a particular spectrometer often demonstrate significantly reduced performance when applied to different instruments, production batches, operators, or environmental conditions [53]. This limitation undermines the industrial scalability and field adoption of the technology.

To mitigate this issue, various calibration transfer strategies have been developed to adapt models across instruments without rebuilding them from scratch. Classical methods such as direct standardisation (DS) and piecewise direct standardisation (PDS) have been fundamental in addressing instrumental variability, while techniques such as EMSC, OSC, or EPO remain widely used in industrial applications [54].

In recent years, advances in deep learning and domain adaptation have led to new approaches based on autoencoders, generative networks, and hybrid spectrum–image models. These models are capable of projecting spectra from different instruments into a shared latent space, thereby improving interoperability.

The validation of spectral models has evolved towards more demanding schemes that reflect the complexity of real-world scenarios. Rather than relying solely on internal partitions (training/testing), the use of external, multi-origin validation is increasingly being promoted. This incorporates samples from various batches, suppliers, seasons, operators, and environmental conditions [55]. This approach enables the robustness, temporal stability, and generalisation capacity to be assessed.

The move towards critical applications, such as food inspection, rapid diagnostics, and official control, requires vibrational spectroscopy-based methods to comply with international standards and regulatory guidelines. Key frameworks include ISO/IEC 17025 for testing laboratories [56], the ICH Q2(R1) analytical validation guideline [57], AOAC guidelines for qualitative and quantitative methods [58], and specific requirements from agencies such as the FDA, EFSA, and EMA [59,60].

In this context, increasing emphasis is placed on the following:

- Metrological traceability, including the use of certified reference materials and standardised protocols [61].
- Comprehensive model documentation detailing preprocessing, variable selection, machine learning architecture, validation criteria, and applicability limits.
- Explainability and auditability of models are particularly important in AI-based systems, where transparency is a prerequisite for regulatory acceptance [62,63].
- Secure data management and cybersecurity are especially relevant for connected portable devices or in-line systems.

The combination of transferable calibration, rigorous validation, and regulatory readiness is fundamental to the transition of vibrational spectroscopy from laboratory to real-world applications. Without these elements, models lack reproducibility, results are unacceptable for official inspection, and the technology cannot be scaled up for industrial use. However, when these aspects are systematically addressed, vibrational spectroscopy becomes a robust, certifiable tool that can be integrated into quality control processes, in-line monitoring, and rapid diagnostics.

## 8. Conclusions and Future Perspectives

Vibrational spectroscopy—particularly NIR, Raman, and FTIR—continues to evolve rapidly, driven by advances in instrumentation, data analytics, and artificial intelligence. The developments reported throughout 2025 have highlighted a decisive shift towards more intelligent, miniaturised, and application-oriented spectroscopic systems. These innovations are transforming the acquisition, processing, and interpretation of spectral information, enabling more robust, autonomous, and scalable analytical workflows across a range of scientific and industrial fields.

A major emerging trend is the deep integration of AI and machine learning into every stage of the spectroscopic pipeline. Recent analyses have demonstrated that AI-powered models now outperform traditional chemometric approaches in tasks such as spectral deconvolution, feature extraction, calibration transfer, and uncertainty quantification. They also enable real-time decision-making in complex environments [64]. These developments are complemented by the emergence of physics-informed neural networks and hybrid simulation–spectroscopy frameworks, which improve interpretability and bridge the gap between empirical spectra and an understanding of the molecular level.

In parallel, the miniaturisation of instruments and the proliferation of portable and handheld spectrometers are expanding the reach of vibrational spectroscopy beyond laboratory settings. Reports from 2025 have emphasised that compact NIR and Raman devices supported by embedded AI algorithms are increasingly capable of delivering a laboratory-grade performance in the field, in clinical settings, and in industrial environments [65]. This trend is expected to accelerate as sensor integration, photonic engineering, and low power electronics continue to mature.

Another key development is the growth of multimodal and data fusion approaches, which combine NIR, Raman, FTIR, hyperspectral imaging, and other complementary sensing modalities. These strategies provide richer chemical and structural information, improve model robustness, and enable more comprehensive characterisation of heterogeneous or dynamic systems [66]. As computational capacity increases, multimodal fusion is likely to become the standard analytical approach rather than a specialised technique.

Looking ahead, the field is set to undergo further transformation. The landscape in 2025 suggests that future progress will be shaped by the following:

- Autonomous spectroscopic systems capable of self-calibration, self-diagnosis, and adaptive measurement strategies;
- Edge AI architectures enabling real-time spectral interpretation directly on portable devices;
- Advanced SERS and photonic platforms offering unprecedented sensitivity and selectivity;
- Standardised, open spectral databases to support reproducibility, model transfer, and large-scale AI training;
- Closer integration of spectroscopy with digital twins and simulation driven design for predictive analytics and process optimisation [67].

Overall, the technological trends described in this review—miniaturisation, enhanced optical architectures, SERS substrates, fibre optic integration, hyperspectral imaging, and advanced chemometrics—are not merely engineering developments, they are the foundation enabling practical, real-time applications in health and food safety control. Portable NIR and Raman devices now support on-site screening and authentication; SERS and microfluidic platforms enable trace level biomarker detection; and HSI systems provide spatially resolved quality and contamination assessment in both clinical and industrial environments. As these tools continue to mature, their integration into routine monitoring, regulatory frameworks, and automated decision-making systems will further strengthen their impact on public health and food safety assurance [68,69].

Table 5 summarises the key strengths, limitations, analytical performance characteristics, and typical application domains of the major techniques discussed in this review.

## Figures and Tables

**Table 1:** Differences and Similarities of Near-Infrared and Raman Spectroscopy.

	NIR	Raman
**Similarities**	Use of molecular vibrations to extract chemical information	
	Non-destructive and fast analysis	
	Used for qualitative and quantitative analysis	
**Differences**	Measures absorption of near-infrared light (typically 700–2500 nm).	Measures inelastic scattering of monochromatic light (usually a laser).
	Broad, overlapping bands due to overtones and combination vibrations.	Sharp, well-resolved peaks corresponding to the fundamental vibrations.
	Strongly affected by water absorption	Water is a weak Raman scatterer
	Minima sample preparation.	Sample preparation is minimal, but lasers may damage sensitive samples due to heating.

NIR: Near-infrared.

**Table 2:** Main specifications of popular miniaturised NIR spectrometers.

Manufacturer/ Headquarters	Model	Technology	Spectral Range (nm)	Spectral Resolution (nm)
Texas Instruments/ Dallas, TX, USA	NIRscan™ Nano EVM	Grating–MEMS DMD	900–1700	10
Viavi Solutions/ Chandler, AZ, USA	Micro-NIR Pro 1700	LVF–Linear	908–1676	12–25
Si-Ware Systems/ Menlo Park, CA, USA	NeoSpectra	MEMS–FT	1250–1700	8–16
Ocean Optics Orlando, Florida, USA	Flame NIR	Grating	970–1700	~10.0
ITPhotonics Fara Vicentino, Vicenza, Italia	Polispec NIR	x	900–1700	3.2
Thermo Fisher Scientific/ Waltham, MA, USA	MicroPHAZIR™	MEMS	1596–2396	11
Southwest Technologies Inc./ North Kansas City, MO, USA	Nano-FTIR	MEMS-Michaelson Interferometer	800–2600	2.5–13
Spectral Engines/ Steinbach, Germany	NIRONE Sensor	MEMS Fabry–Pérot Interferometer	1350–2500	16

**Table 3:** Main specifications of popular Raman spectrometers.

Model/ Manufacturer	Laser Wavelength	Laser Power	Spectral Range (cm^−1^)	Spectral Resolution
Pendar X10/ Pendar Technologies LLC, USA	~830 nm Difference Raman Spectroscopy	Max. 90 mW	Measures from a distance from 0.3 to 2 m	X
BRAVO/ Bruker Optics	Laser Duo: 785 nm and 852 nm	Class 1 Laser	~170 cm^−1^–~2.200 cm^−1^	10–12 cm^−1^
B&W Tek i-Raman Plus/ Metrohm AG	532 nm or 785 nm	~340 mW (785 nm)	~65 cm^−1^–~4200 cm^−1^	<~4.5 cm^−1^
miniRaman/ Lightnovo	Standard dual: 785 nm, 660/675 nm	5–50 mW; 5–40/5–75 mW	2336–460 cm^−1^	2 cm^−1^
NanoRam-1064/ Metrohm AG	1064 nm	420 ± 30 mW	176–2500 cm^−1^	~10–11 cm^−1^

**Table 4:** Comparative table of the most widely used chemometrics tools.

Name/Type	Key Features (Short)	Strengths	Weaknesses	Typical Users
**PLS_Toolbox/Solo (Eigenvector)/** Commercial (MATLAB add-on)	PLS, PCR, multiway (PARAFAC/Tucker), preprocessing, validation, many visualisation tools.	Very feature-rich for spectroscopy & calibration; tight MATLAB integration; mature.	Requires MATLAB (unless using Solo); commercial cost.	Analytical chemists, spectroscopists, model developers.
**The Unscrambler X (CAMO/AspenTech)/** Commercial	PCA, PLS, calibration, process monitoring, DoE, SPC, strong visualisation.	GUI-driven, industry-proven for spectroscopy and PAT; lots of built-in workflows.	Commercial cost; less code/scriptable than Python/R.	Industrial spectroscopy labs, process analytics teams.
**SIMCA (Sartorius/Umetrics^®^)/** Commercial	Multivariate process monitoring, PCA/PLS/SIMCA models, real-time monitoring.	Built for process control/PAT and real-time batch monitoring; strong automation and deployment.	Commercial; enterprise focus can be heavyweight for small projects.	Biopharma/process industries, PAT teams.
**Pirouette (Infometrix)/** Commercial	PCA, PLS, classification, mixture analysis, automation tools.	Fast, focused chemometrics GUI; long history in spectroscopy communities.	Smaller vendor; fewer ecosystem integrations than MATLAB/Python.	QC labs, spectroscopy analysts who want GUI workflows.
**Mnova—Advanced Chemometrics (Mestrelab)** Commercial (plugin/module)	PCA, PLS, SIMCA, MCR-ALS, spectral preprocessing integrated with NMR/LC/MS workflows.	Excellent if you already use Mnova for spectra—combines spectral processing + chemometrics in one place.	Requires Mnova license; more specialised toward NMR/LC-MS users.	Spectroscopists, NMR/LC-MS users who want integrated workflow.
**OPUS (Bruker)/** Commercial	Spectrometer control + spectral processing + multivariate analysis extensions.	Industry-leading spectroscopy software with instrument integration and reaction monitoring plugins.	Mainly tied to Bruker instruments; commercial.	FT-IR/Raman/NIR users using Bruker instruments.
**TQ Analyst/OMNIC (Thermo Fisher)/** Commercial	Spectral chemometrics (PCR/PLS), spectral libraries, method dev & deployment.	Designed for spectroscopists and manufacturing deployment; vendor support for regulatory environments.	Commercial; tied to Thermo ecosystem for full integration.	QA/QC labs, spectroscopy method developers.
**mdatools (R package)**	PCA, PLS, preprocessing, validation, visualisation, tutorials & docs.	Free, actively maintained, good for reproducible scripting and teaching.	Requires R programming; GUI options limited.	Academics, students, data scientists preferring R.
**chemometrics (Python package)/** **Open source**	Spectral preprocessing, plotting, PLS, PCA—built on scikit-learn.	Python-friendly, integrates with scikit-learn ecosystem for ML + chemometrics.	Smaller feature set vs. mature commercial suites; requires coding.	Data scientists & researchers who prefer Python.
**pyChemometrics/chemotools/scikit-spectra (Python eco.)/** Open source	PCA/PLS wrappers, spectral transformers, scikit-learn integration, spectroscopy datatypes.	Flexible, ideal for building reproducible pipelines; many specialised transformers available.	Fragmented ecosystem (multiple small packages); need to assemble pipeline.	Python users building production or research pipelines.

**Table 5:** Comparative assessment of vibrational spectroscopy techniques for food and medical applications.

Technique	Key Strengths	Limitations	Typical Analytical Performance	Suitability for Food Applications	Suitability for Medical/Clinical Applications
**Near-Infrared (NIR) Spectroscopy**	Rapid, non-destructive; Deep penetration in biological matrices; Minimal sample prep.	Broad, overlapping bands; Lower chemical specificity.	Good for quantitative models; Moderate sensitivity.	Moisture, protein, fat quantification; Process monitoring (PAT).	Tissue characterisation; Non-invasive metabolic screening.
**Mid-Infrared (MIR) Spectroscopy**	High molecular specificity; Strong fundamental vibrations.	Limited penetration depth; Sensitive to water.	High specificity; Excellent qualitative profiling.	Authentication, adulteration detection; Structural characterisation.	Biofluid analysis (serum, saliva); Disease biomarker detection.
**Raman Spectroscopy**	High chemical specificity; Water-insensitive; Compatible with in vivo probes.	Fluorescence interference; Lower scattering efficiency.	High specificity; Good for structural fingerprints.	Detection of contaminants, adulterants; Quality grading of fruits, oils.	Cancer diagnostics; In vivo tissue analysis; Point-of-care probes.
**Hyperspectral Imaging (HSI)**	Combines spatial + spectral data; Enables mapping of heterogeneity.	Large data volumes; Requires advanced chemometrics.	High spatial resolution; Good classification accuracy.	Surface quality, defects, ripeness Sorting and grading	Wound assessment Tissue oxygenation mapping
**Surface-Enhanced Raman Spectroscopy (SERS)**	Ultra-high sensitivity; Suitable for trace detection.	Reproducibility challenges; Substrate variability.	Very high sensitivity; Suitable for trace biomarkers.	Detection of pesticides, toxins; Trace contaminants.	Biomarker detection; Liquid biopsy approaches.

## Data Availability

Data will be made available upon request.
